# Single‐cell analysis reveals the multiple patterns of immune escape in the nasopharyngeal carcinoma microenvironment

**DOI:** 10.1002/ctm2.1315

**Published:** 2023-06-22

**Authors:** Qianyu Lin, Yaqi Zhou, Jie Ma, Sanyang Han, Yunchuanxiang Huang, Feng Wu, Xuejuan Wang, Yanan Zhang, Xueshuang Mei, Lan Ma

**Affiliations:** ^1^ Tsinghua‐Berkeley Shenzhen Institute Tsinghua University Beijing China; ^2^ Department of Otorhinolaryngology Peking University Shenzhen Hospital Shenzhen China; ^3^ Department of Radiology Shenzhen People's Hospital Shenzhen China; ^4^ Tsinghua Shenzhen International Graduate School Tsinghua University Beijing China; ^5^ Shenzhen Bay Laboratory Shenzhen China

## Abstract

**Background:**

Single‐cell transcriptomics has revolutionised our understanding of the cellular composition of the tumour microenvironment (TME) in nasopharyngeal carcinoma (NPC). Despite this progress, a key limitation of this technique has been its inability to capture epithelial/tumour cells, which has hindered further investigation of tumour heterogeneity and immune escape in NPC.

**Methods:**

In this study, we aimed to address these limitations by analysing the transcriptomics and spatial characteristics of NPC tumour cells at single‐cell resolution using scRNA/snRNA‐seq and imaging mass cytometry techniques.

**Results:**

Our findings demonstrate multiple patterns of immune escape mechanisms in NPC, including the loss of major histocompatibility complex (MHC) molecules in malignant cells, induction of epithelial–mesenchymal transition in fibroblast‐like malignant cells and the use of hyperplastic cells in tumour nests to protect tumour cells from immune infiltration. Additionally, we identified, for the first time, a CD8+ natural killer (NK) cell cluster that is specific to the NPC TME.

**Conclusions:**

These findings provide new insights into the complexity of NPC immune landscape and may lead to novel therapeutic strategies for this disease.

## Graphical abstract

1. NPC malignant cells exhibit heterogeneity in MHC expression.
2. Fibroblast‐like malignant cells demonstrate enhanced immunoevasive capabilities.
3. NPC tumour nests utilise hyperplastic cells to isolate themselves from immune infiltration.

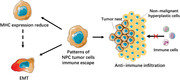

## 1 INTRODUCTION

Nasopharyngeal carcinoma (NPC) is a prevalent type of head and neck cancer in Southeast Asia and is commonly associated with Epstein‒Barr virus (EBV) infection.^1^, ^2^, ^3^ NPC has a high incidence of metastasis (15%−30%) and recurrence (41%).^4^ These unfavourable outcomes may be attributed to the immune escape behaviour of malignant cells. Single‐cell technology has the potential to shed light on the heterogeneity of malignant cells in the NPC tumour microenvironment (TME) with regard to immune escape features. However, previous studies using single‐cell RNA sequencing failed to capture epithelial/tumour cells efficiently, thereby impeding a comprehensive analysis of NPC malignant cell heterogeneity.^5^, ^6^, ^7^, ^8^

In this study, we aimed to investigate the heterogeneous immune escape behaviours of NPC in its TME. The immune escape in tumours refers to the ability of tumour cells to evade the recognition and immune response from the host immune system, which is a significant factor in cancer therapy failure.^9^ Common ways by which tumour cells achieve immune escape include MHC molecule loss and immune checkpoint inhibitor expression.^10^, ^11^ To investigate the single‐cell heterogeneity of NPC malignant cells from a transcriptomic perspective, as well as the spatial distribution characteristics of malignant cells at single‐cell resolution, we used single‐cell multiomics technology on clinical biopsy NPC tissue samples. We sequenced 50 439 single cells and 62 003 single nuclei, and identified 670 044 single cells from imaging mass cytometry (IMC) images. Our analysis uncovered various patterns of immune escape behaviours in NPC, including the loss of MHC molecules in malignant cells, enhanced immune escape capacity in epithelial–mesenchymal transition (EMT)‐induced fibroblast‐like malignant cells and utilisation of hyperplastic cells to protect tumour cells from immune cell infiltration. Additionally, we identified a CD8+ NK cell cluster specific to the NPC TME with enhanced antiviral functions, which had not been previously described. In conclusion, our research provides new insights into the heterogeneity of malignant cells in situ in NPC regarding immune escape.

## 2 RESULTS

### 2.1 The multiomics atlas of NPC TME

**FIGURE 1:**
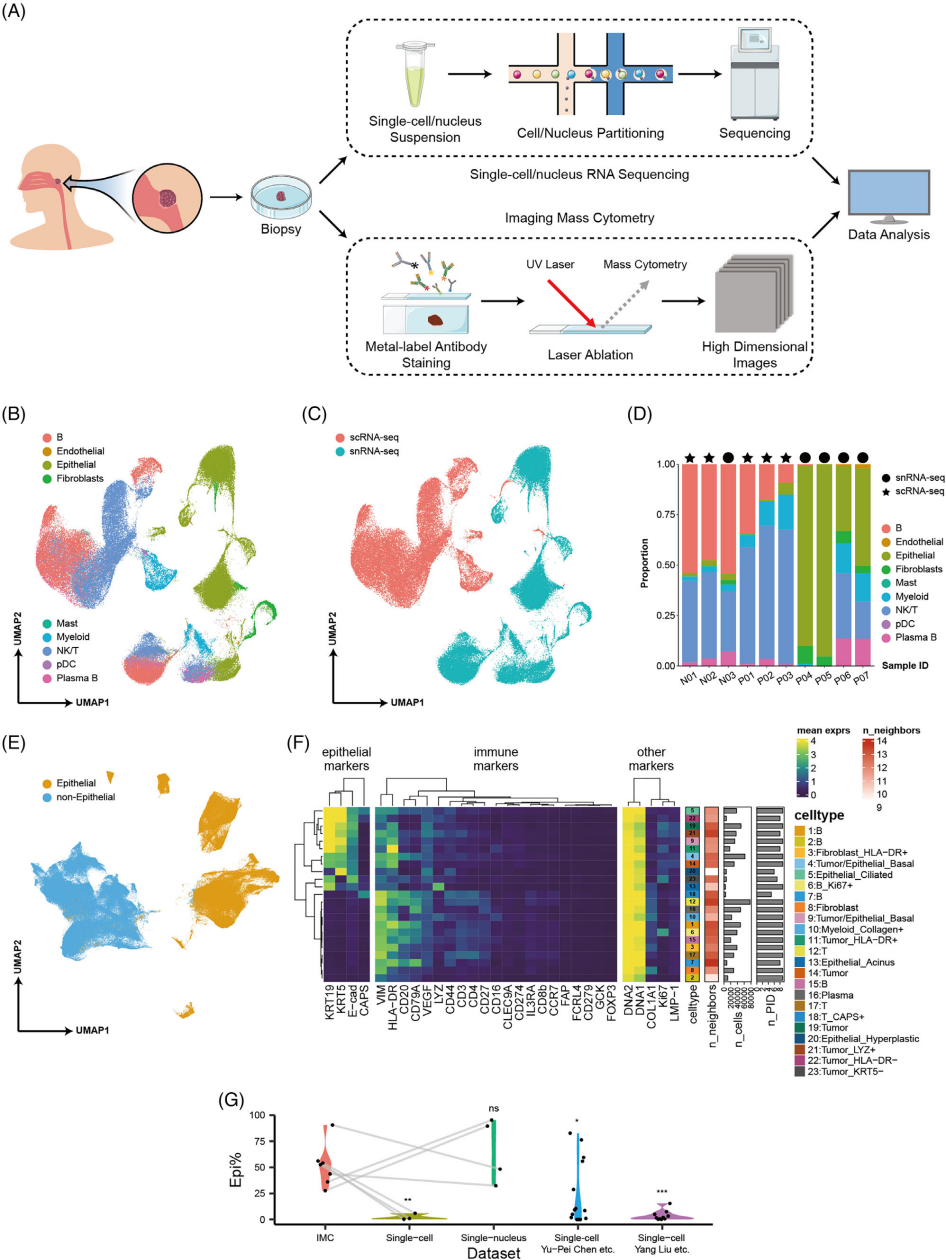
The multiomics single‐cell atlas of nasopharyngeal carcinoma (NPC) obtained by scRNA/snRNA‐seq and imaging mass cytometry (IMC) imaging. (a) The workflow of the multiomics single‐cell analysis of NPC tumour microenvironment (TME), including tissue collection, scRNA/snRNA sequencing, IMC imaging and data analysis. (b) UMAP plot of all single cells obtained in scRNA/snRNA‐seq coloured by nine cell types. (c) UMAP plot of all single cells obtained in scRNA/snRNA‐seq coloured by sequencing strategies. (d) Cell composition of 10 sequenced samples in scRNA/snRNA‐seq data. (e) UMAP plot of all single cells obtained in IMC images coloured by epithelial/non‐epithelial groups. (f) The integrated information visualisation of each annotated cell type, including the mean marker expression, mean number of neighbours, number of cells and number of contributing patients per cell type. (g) Violin plot of the comparison of epithelial cell proportions between IMC images, scRNA‐seq data, snRNA‐seq data and other accessible scRNA data. Statistical analysis was performed using a one‐sided Wilcoxon test: ^*^ *p* < 0.05; ^**^ *p* < 0.01; ^***^ *p* < 0.001; ns, the hypothesis (the epithelial cell proportion in the test group is less than that in the IMC group) is not valid.

In this study, we collected three non‐malignant tissues and seven NPC samples from nine donors via clinical biopsy, from which the transcriptomics atlas and proteomic atlas were generated by using scRNA/snRNA sequencing and IMC imaging technologies (Figures 1A and S1A).

Given the differential capture efficiency of scRNA and snRNA sequencing technologies for epithelial and immune cells, both sequencing methods were used in this study to capture a wide range of cell types in the NPC TME. Specifically, scRNA‐seq was applied to two non‐malignant tissues (N01, N02; from the same patient) and three NPC tissues (P01, P02, P03), while snRNA‐seq was used for one non‐malignant tissue (N03) and four NPC tissues (P04, P05, P06, P07). Following mapping the sequencing reads on the EBV‐integrated human genome reference and quality control (removing low‐quality cells and doublets), a total of 50 439 single cells and 62 003 single nuclei were retained for downstream analysis. Cell subtype annotation was performed separately for each sample dataset, taking into account the variations due to tumour heterogeneity and sequencing methods. In total, we identified nine main cell types in the NPC TME, including B cells (*CD79A/B*+, *MS4A1*+), endothelial cells (*FLT1*+, *VWF*+), epithelial cells (*KRTs*+), fibroblasts (*COL1A1*+), mast cells (*TPSAB1*+), myeloid cells (*LYZ*+), NK/T cells (*CD3D/E*+, *GNLY*+), plasmacytoid dendritic cells (*IL3RA*+) and plasma B cells (*CD79A/B*+, *MS4A1–*, *IGs*+) (Figures 1B–D and S1B).

Using the IMC technique, we characterised a spatially resolved TME of NPC by analysing the paraffin sections from nine patients with a 28‐marker antibody panel covering 53 regions of interest (ROIs). We identified 670 044 single cells and clustered them into 23 clusters (Figures 1E–G and S1C,D). The majority of the cell clusters were annotated based on cell type markers, such as B cells (*CD20*+, *CD79A*+), plasma B cells (*CD20–*, *CD79A*+), T cells (*CD3*+), myeloid cells (*LYZ*+), epithelial cells (*KRT5*+ or *KRT19*+) and fibroblasts (*COL1A1*+). Epithelial cell clusters, including ciliated epithelial cells, basal epithelial cells, acinus and tumour cells, were further annotated based on both markers and cell morphology. However, due to the overly high cell density of the tumour nest and the limited IMC resolution, some adjacent tumour cells and infiltrated immune cells were recognised as epithelial–immune dual feature cells, such as Clusters 4 (*KRT5*+, *KRT19*+, *CD20*+ and *CD79A*+) and 14 (*KRT5*+, *KRT19*+ and *LYZ*+). These cells were identified as a result of the cell segment strategy, which caused the membrane surface proteins originating from one side of an adjacent cell boundary to be shared by both sides of the cells. These clusters reflect the abundant immune cells infiltrating the tumour nest.

The cellular composition of scRNA/snRNA data was found to vary among samples in this study. It was observed that scRNA sequencing samples had a significantly smaller proportion of epithelial cells than the snRNA sequencing samples (Figure 1D). These findings are in line with the results of previous studies on the variability of single‐cell sequencing techniques.^12^ It was also noted that the underestimation of epithelial cells was a common feature of other NPC scRNA studies.^5^, ^6^, ^7^, ^8^ Although NPC is known to exhibit abundant immune cell infiltration, the proportion of epithelial cells in scRNA‐seq data was observed to be too low to be consistent with the clinical observations of haematoxylin and eosin (HE)‐stained sections. In comparing the NPC sample sourced epithelial cell proportion in different datasets, the results indicated that the scRNA method significantly underestimated the proportion of epithelial cells in the NPC TME (Figure 1G). While two NPC scRNA datasets were not included in the comparison, the underestimation of epithelial cells was still evident in their results. The Gong et al. dataset showed that the epithelial cell proportion in all NPC samples was too small to be statistically significant^8^; in the Jin et al. dataset, almost all epithelial cells were predominantly from the two NPC samples sequenced out of 17 samples. Overall, snRNA‐seq was found to be more efficient in capturing epithelial cells and therefore, it was concluded that snRNA‐seq is a better choice for analysing the transcriptomic heterogeneity of tumour cells in the NPC TME than scRNA‐seq.

### 2.2 Tumour heterogeneity of malignant cells in the NPC TME

**FIGURE 2:**
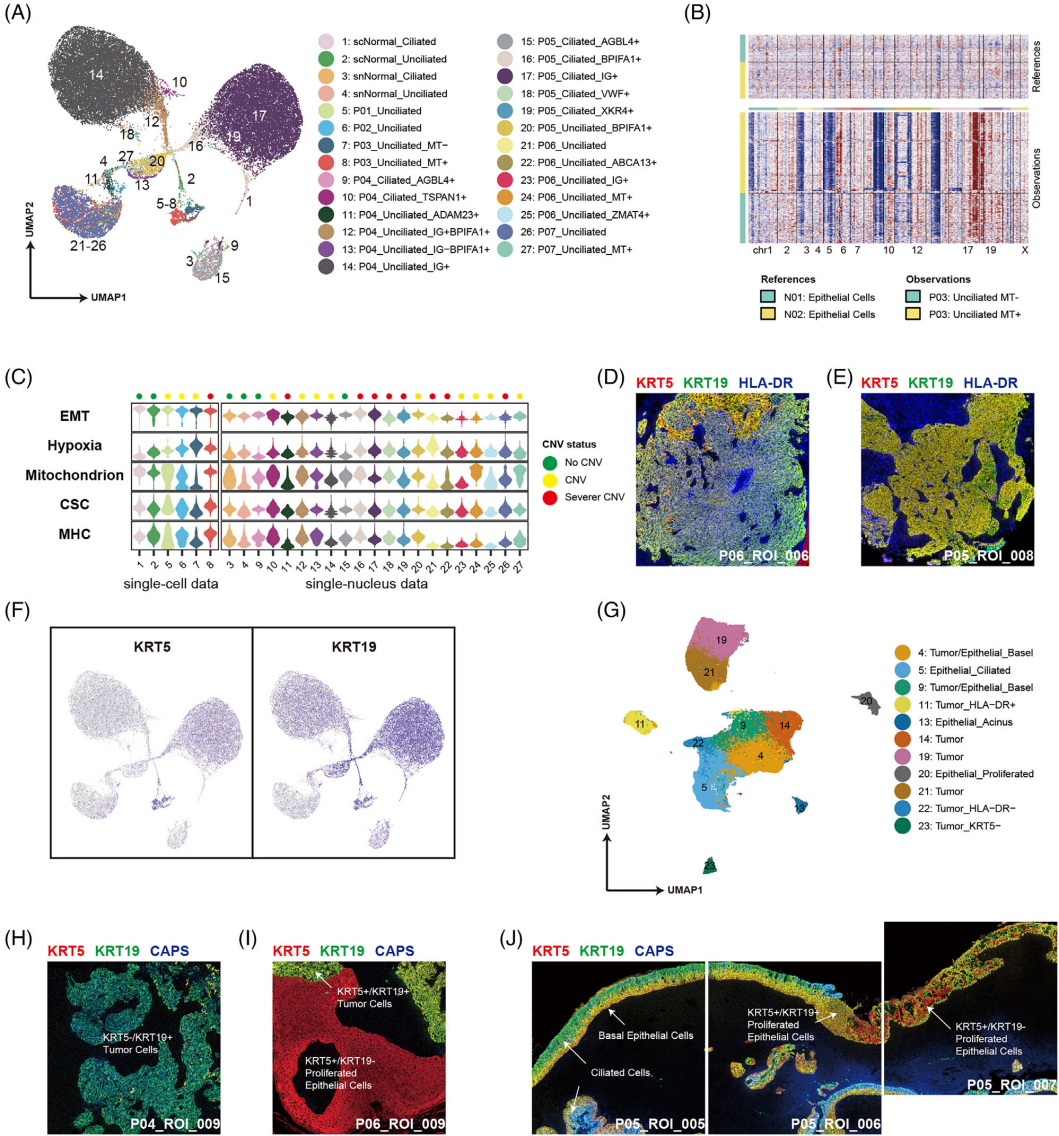
Heterogeneity of epithelial cells in the normal nasopharyngeal microenvironment and nasopharyngeal carcinoma (NPC) tumour microenvironment (TME). (a) UMAP plot of all epithelial cells coloured by cell cluster annotation on transcriptomics data. (b) Copy number variation (CNV) inference result of epithelial cells in the P03 sample, by using epithelial cells from normal samples (N01, N02) as reference. (c) Violin plots of epithelial–mesenchymal transition (EMT), hypoxia, mitochondria, tumour stem and MHC scores for each epithelial cell cluster, with the numbers in the horizontal coordinates being the number of the cell cluster consistent with the annotation of (a). The CNV status of each cell cluster is marked at the top of the graph (green: no CNV; red: CNV). (d and e) Tumour cells with differential expression of HLA‐DR antigen. Imaging mass cytometry (IMC) images numbered P06_ROI_006 and P05_ROI_008. Pseudostaining for KRT5 (red), KRT19 (green) and HLA‐DR (blue) in cells. Each image size is 1 mm square. (f) The expression of KRT5 and KRT19 in each epithelial cell profiled on the UMAP plots. (g) UMAP plot of all epithelial cells coloured by cell cluster annotation on IMC data. (h and i) Epithelial cells with differential expression of KRT5 and KRT19. IMC images numbered P04_ROI_009 and P06_ROI_009. Pseudostaining for KRT5 (red), KRT19 (green) and CAPS (blue) in cells. Representative epithelial cells are indicated by white arrows with labels. Each image size is 1 mm square. (j) The process of alienation of pseudostratified columnar epithelial cells from a normal structure to a dedifferentiation state. Consecutive IMC images numbered P05_ROI_005, P05_ROI_006 and P05_ROI_007. Pseudostaining for KRT5 (red), KRT19 (green) and CAPS (blue) in cells. Representative epithelial cells are indicated by white arrows with labels. Each image size is 1 mm square.

We focused on epithelial cells and completely annotated 27 epithelial cell clusters, naming them based on their sample origin, differentially expressed genes (DEGs) and cilia status (marked by *CAPS, RSPH1*). The results of the annotation are presented in a UMAP plot that includes all epithelial cells (Figure 2A). In non‐malignant samples, we observed only two epithelial cell types: ciliated epithelial cells and unciliated basal epithelial cells, which reflects the pseudostratified columnar epithelial structure in normal nasopharyngeal tissue. In contrast, epithelial cells in NPC samples that have lost their cilia displayed a high degree of heterogeneity, indicating a dysregulated mechanism of differentiation of ciliated cells in tumour cells. Our IMC images show that tumour cells in the TME do not necessarily lose ciliary gene expression completely (Figure 1F). However, the correlation between the degree of ciliation and the prognosis of NPC remains unclear.

One of the most distinguishing features of malignant cells from non‐malignant cells is their abnormality in copy number variation (CNV), which is the criterion used to identify malignant cells in this study. The loss or duplication of the genes located on a contiguous segment of a chromosome is defined as a chromosomal abnormality. The results of CNV inference demonstrated that at least two phenotypes were present in every NPC sample in which a valid number of epithelial cells were captured (*n* > 100), as illustrated in Figure S2A–A. Using P03 as an example (Figure 2B), is was observed that Clusters 7 (P03 Unciliated MT–) and 8 (P03 Unciliated MT+) exhibited distinct CNV phenotypes, with cells in Cluster 8 demonstrating more severe chromosome loss in chr1, 3, 4, 9, 11 and 14, as well as more pronounced chromosome duplication in chr6, 17 and 18 compared to those in Cluster 7. Furthermore, Cluster 8 also contained two other epithelial cell phenotypes, namely, non‐CNV epithelial cells and malignant cells exhibiting loss of chr3, 4, 8, 9, 10, 14 and 16. Our CNV inference results suggest that NPC malignant cells with different CNV phenotypes coexist within the NPC TME, which highlights the tumour heterogeneity of NPC malignant cells.

To expand our investigation of the heterogeneity of malignant cells, we examined additional module functions of epithelial cells regarding EMT, hypoxia, mitochondrial activity, stemness, and MHC molecule expression. We visualised the results as violin plots, which can be found in Figure 2C. Malignant cells positive for keratin exhibited EMT scores that were more similar to those of non‐malignant cells, suggesting that these cells still retained their epithelial cell polarity. Hypoxia is frequently observed in the TME and has been linked to malignant behaviours in various tumour types, including therapy resistance and metastasis.^13^, ^14^ We observed higher hypoxia scores in malignant cell clusters than in non‐malignant epithelial cells in normal nasopharyngeal tissue or NPC samples, suggesting that hypoxia is widespread in the tumour region. In addition, we identified increased mitochondria scores in certain malignant cell clusters, such as Clusters 8, 12, 24 and 27. However, we did not observe any significant correlation between hypoxia scores and mitochondria scores (Pearson correlation coefficient, *r* = 0.02), indicating that mitochondrial transcript levels remain stable in NPC malignant cells under hypoxic conditions. The stemness scores were calculated based on cancer stem cell (CSC) markers,^15^ and exhibited variations among different cell clusters within the same sample. Additionally, the expression level of MHC molecules reflects the antigen presentation ability of the cells, and the low expression of MHC molecules in malignant cells is associated with immune escape.^10^ We observed that certain malignant tumour clusters, such as Clusters 7 and 11, exhibited low MHC scores, indicating a potentially higher immune escape ability. Additionally, the IMC images revealed differential expression of MHC molecules (human leukocyte antigen – DR [HLA‐DR]) in malignant cells (Figure 2D,E), and malignant cells with low MHC expression exhibit stronger proliferation ability (Figure S2E).

Besides the varied expression of markers indicative of malignant tumour behaviour in malignant cells, our study also revealed distinct expression patterns of different keratins, namely, *KRT5* and *KRT19*, at the transcriptome level in epithelial cells (Figure 2F). Consequently, we employed *KRT19* as a marker in IMC imaging. Epithelial cell annotation in IMC images was visualised in a UMAP plot (Figure 2G), wherein differential expression of KRT19 was also observed across distinct epithelial cell clusters. Interestingly, contrary to the transcriptomic findings, *KRT5* and *KRT19* were coexpressed in most tumour cells and normal basal epithelial cells in IMC images (Figure 1F), except for a subset of KRT5–/KRT19+ tumour cells in P04 (Figure 2H). In non‐malignant hyperplastic epithelial cells, *KRT19* was generally negative (Figure 2I). However, hyperplastic epithelial cells located at the periphery of the hyperplasia regions displayed enhanced cell proliferation functions, as demonstrated by their *Ki67+* phenotype (Figure S2F–H). Additionally, consecutive ROIs from P05 illustrated the transformation of pseudostratified columnar epithelial cells from a normal state to a proliferative state: *KRT19+* ciliated cells were replaced by hyperplastic *KRT5+/KRT19+* basal epithelial cells, which subsequently progressed into *KRT5+/KRT19*– hyperplastic epithelial cells (Figure 2J). A thickened layer of giant hyperplastic non‐malignant epithelial cells was observed in both the P06 and P07 samples (Figures S1A and 2I).

### 2.3 Fibroblast‐like malignant cells exhibit a greater propensity for immune escape than epithelial‐like malignant cells

**FIGURE 3:**
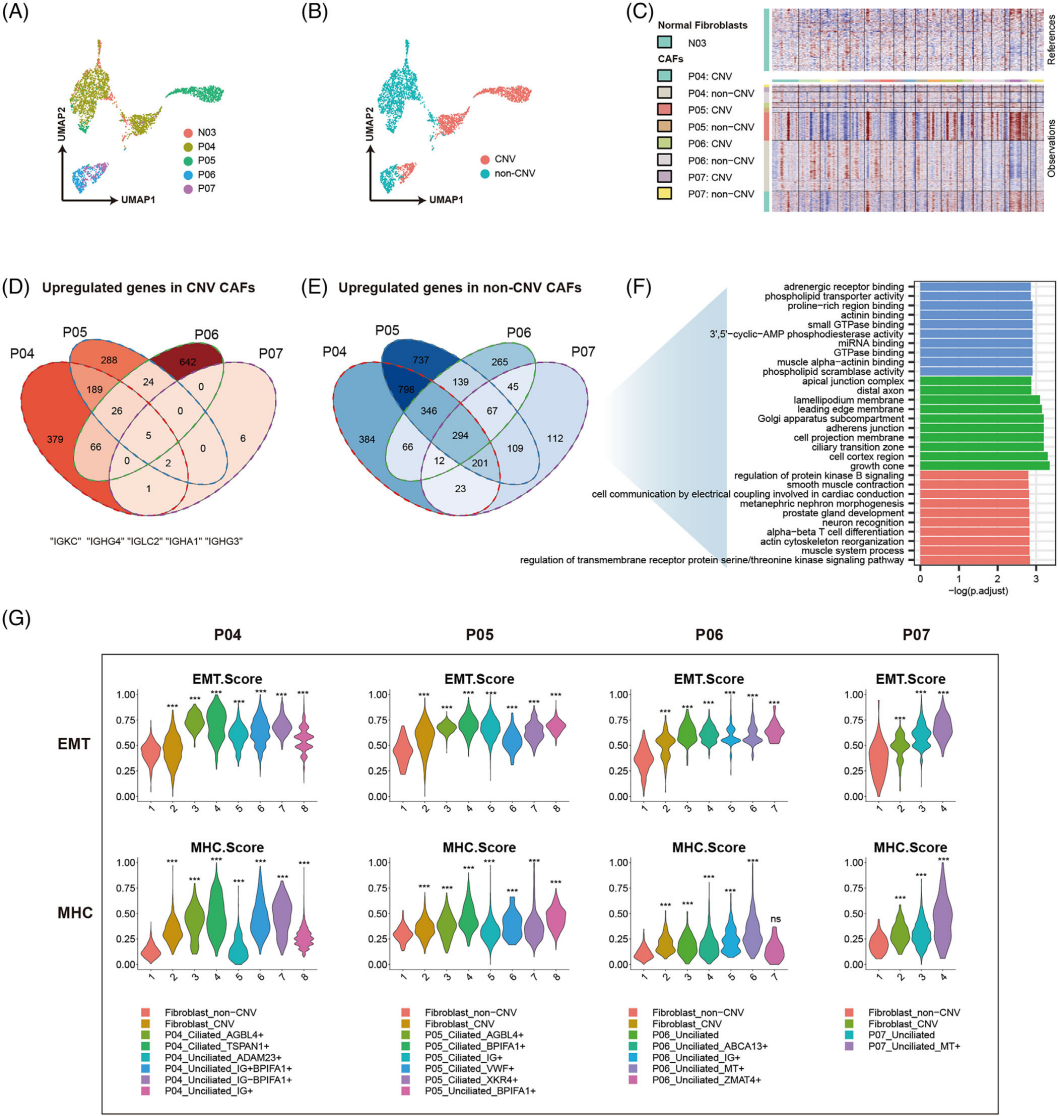
Heterogeneity of fibroblasts in the normal nasopharyngeal microenvironment and nasopharyngeal carcinoma (NPC) tumour microenvironment (TME). (a and b) UMAP plot of all fibroblasts coloured by cell sample source and cell copy number variation (CNV) status. (c) CNV inference result of all fibroblasts derived from NPC samples, by using fibroblasts from normal samples (N03) as the reference. (d) The Venn diagram of upregulated genes in fibroblast‐like malignant cells. (e) Venn diagram of upregulated genes in non‐malignant cancer‐associated fibroblasts (CAFs). (f) Bar plots show Gene Ontology (GO) enrichment analysis results for upregulated genes in non‐malignant CAFs. Bars are coloured according to the sub‐ontologies of the pathways (red: biological process; green: cellular component; blue: molecular function). (g) Violin plots of epithelial–mesenchymal transition (EMT) and MHC scores for malignant cell clusters in NPC samples P04 to P07. Statistical analysis was performed using *t* test: ^*^ *p* < 0.05; ^**^ *p* < 0.01; ^***^ *p* < 0.001; ns, the hypothesis (the EMT/MHC score in the test group is higher than that in the control group) is not valid.

Fibroblasts in scRNA/snRNA data were annotated using the marker gene *COL1A1*. Similar to epithelial cells, the scRNA‐seq technique was proved to be highly inefficient in capturing fibroblasts in normal nasopharyngeal and NPC tissues, as also demonstrated in other NPC scRNA studies.^5^, ^6^, ^7^, ^8^ In fact, we were not able to capture fibroblasts with significant cell numbers (*n* > 100) in scRNA‐seq samples. Therefore, all fibroblasts used for downstream analysis were obtained exclusively from snRNA‐seq samples (Figure 3A).

Although cancer‐associated fibroblasts (CAFs) are considered non‐malignant cells, we inferred CNV in these cells using fibroblasts from a normal tissue sample (N03) as the reference. Surprisingly, fibroblast‐like malignant cells were identified in each fibroblast‐accessible NPC sample (Figure 3B,C). We analysed the DEGs between these fibroblast‐like malignant cells and non‐malignant CAFs in each NPC sample. The Venn diagram shows the common genes that were upregulated/downregulated in all NPC samples (Figure 3D,E). Five genes, including *IGKC*, *IGHG4*, *IGLC2*, *IGHA1* and *IGHG3*, which are all immunoglobin (Ig) genes, were upregulated in fibroblast‐like malignant cells. Igs expressed in tumour cells, known as cancer‐derived Igs, have been identified in many cancers, including NPC, and may be associated with promoting malignant behaviour and immune escape in tumour cells.^16^ Enrichment analysis revealed that non‐malignant CAFs upregulated pathways associated with recombinant extracellular matrix (ECM), such as ‘actin cytoskeleton reorganisation’, ‘adherens junction’ and ‘actinin binding’ (Figure 3F).

Next, we aimed to investigate the relationship between fibroblast‐like malignant cells and malignant epithelial cells by comparing their CNV phenotypes. Taking the P04 sample as an example (Figure S3A), fibroblast‐like malignant cells shared the same CNV phenotype as the malignant epithelial cell clusters ‘P04_Ciliated_TSPAN1+’, ‘P04_Unciliated_IG+’ and ‘P04_Unciliated_IG+BPIFA1+’. This phenomenon of fibroblast‐like malignant cells and malignant epithelial cells from the same sample sharing the same CNV phenotype was observed in each snRNA‐seq NPC sample (Figure S3A–D), suggesting that these malignant cells share the common ancestors. The EMT process is a possible way to generate fibroblast‐like malignant cells and it is therefore necessary to investigate the cell polarity of malignant cells. We investigated the cell polarity of malignant cells by calculating epithelial polarity and mesenchymal polarity scores. The EMT score of each cell was defined as its directed distance to the ‘polarisation boundary line’, where the cells on this line had the same scaled epithelial and mesenchymal scores (Figure S3E). The EMT score of each cell was scaled from 0 to 1 and is shown in the violin plot (Figure 3G). Fibroblast‐like malignant cells had higher EMT scores than non‐malignant CAFs, but their EMT scores were still lower than those of most malignant epithelial cell clusters, indicating that their epithelial polarity was not completely lost. We also calculated MHC scores (Figure 3G), with fibroblast‐like malignant cells having lower MHC scores than most malignant epithelial cell clusters, suggesting reduced antigen presentation and enhanced immune escape in fibroblast‐like malignant cells. In summary, fibroblast‐like malignant cells were found in all snRNA‐seq NPC samples and exhibited more malignant features than malignant epithelial cells in terms of Igs expression, EMT features, and immune escape, which may be the potentially malignant cells with high migration capacity, and may be the potential CSCs of NPC. However, we acknowledge that these conclusions are tentative and further research is needed to substantiate these claims.

### 2.4 Non‐malignant hyperplastic areas reduce the immune pressure on the tumour nests

We identified a third pattern by which tumour cells evade the immune system, in addition to reducing MHC antigen presentation and transferring to fibro‐like malignant cells, by physically isolating themselves from immune cells using non‐malignant hyperplastic cells.

**FIGURE 4:**
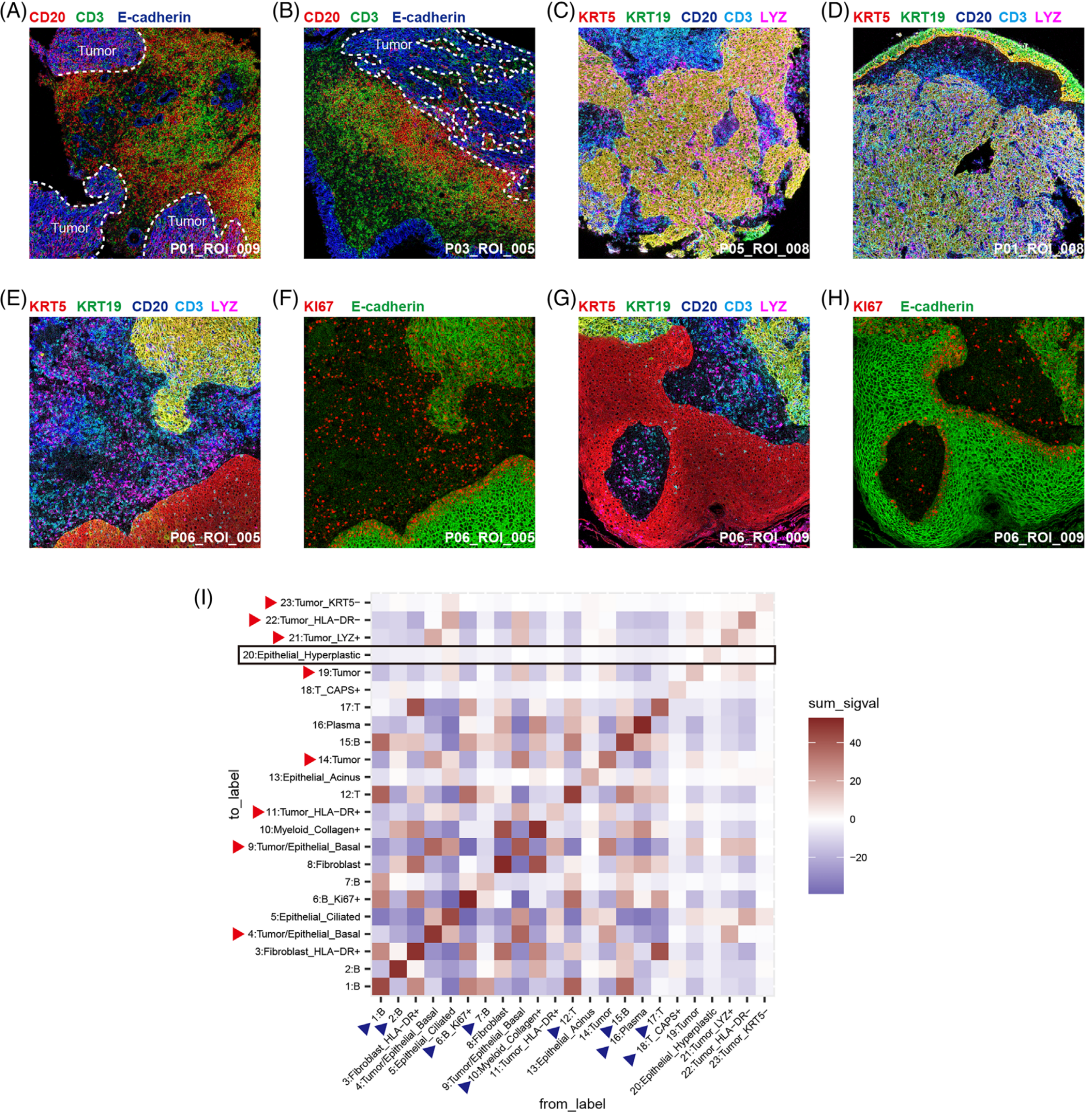
Differential immune infiltration in the nasopharyngeal carcinoma (NPC) tumour microenvironment (TME). (a and b) Infiltration of tumour nests by lymphocytes in the NPC TME. Pseudostaining for CD20 (red), CD3 (green) and E‐cadherin (blue) in cells. The boundaries of the tumour nest are traced with white dashed lines. Each image size is 1 mm square. (c and d) Infiltration of tumour nests by different types of immune cells in the NPC TME. Pseudostaining for KRT5 (red), KRT19 (green), CD20 (blue), CD3 (cyan) and LYZ (pink) in cells. Each image size is 1 mm square. (e) Unadjacent tumour nest and hyperplastic areas in the NPC TME. Pseudostaining for KRT5 (red), KRT19 (green), CD20 (blue), CD3 (cyan) and LYZ (pink) in cells. (f) Pseudostaining for KI67 (red) and E‐cadherin (green) in cells. Each image size is 1 mm square. (g) Adjacent tumour nest and hyperplastic areas in the NPC TME. Pseudostaining for KRT5 (red), KRT19 (green), CD20 (blue), CD3 (cyan) and LYZ (pink) in cells. (h) Pseudostaining for KI67 (red) and E‐cadherin (green) in cells. Each image size is 1 mm square. (i) Heatmap for inferred cell–cell interactions (CCIs) between different cell clusters in imaging mass cytometry (IMC) data. Highlight the Epithelial_Hyperplastic row with a black square, and emphasise malignant cell clusters and immune cell clusters using red triangles and blue triangles, respectively.

NPC is known for its abundant immune infiltration, as depicted in Figure 4A–D, which shows a strong immune response in the NPC TME. However, immune infiltration is heterogeneous in different structures, and there were rare myeloid cells and lymphocytes infiltrating the hyperplastic epithelium regions (Figure 4E,G,I). Once the tumour nest and the hyperplastic areas were adjacent, the immune cells could no longer pass through the hyperplastic epithelium regions and infiltrate the tumour nest. Moreover, hyperplastic epithelial cells were only Ki67+ when adjacent to non‐tumour nest areas (Figure 4F,H). This characteristic made hyperplastic epithelial cells the pioneer and accomplice in tumour nest expansion, as the tumour nest could invade the hyperplastic epithelial regions with less immune pressure.

### 2.5 CD8+ NK cells: the unique cell cluster in the NPC TME

**FIGURE 5:**
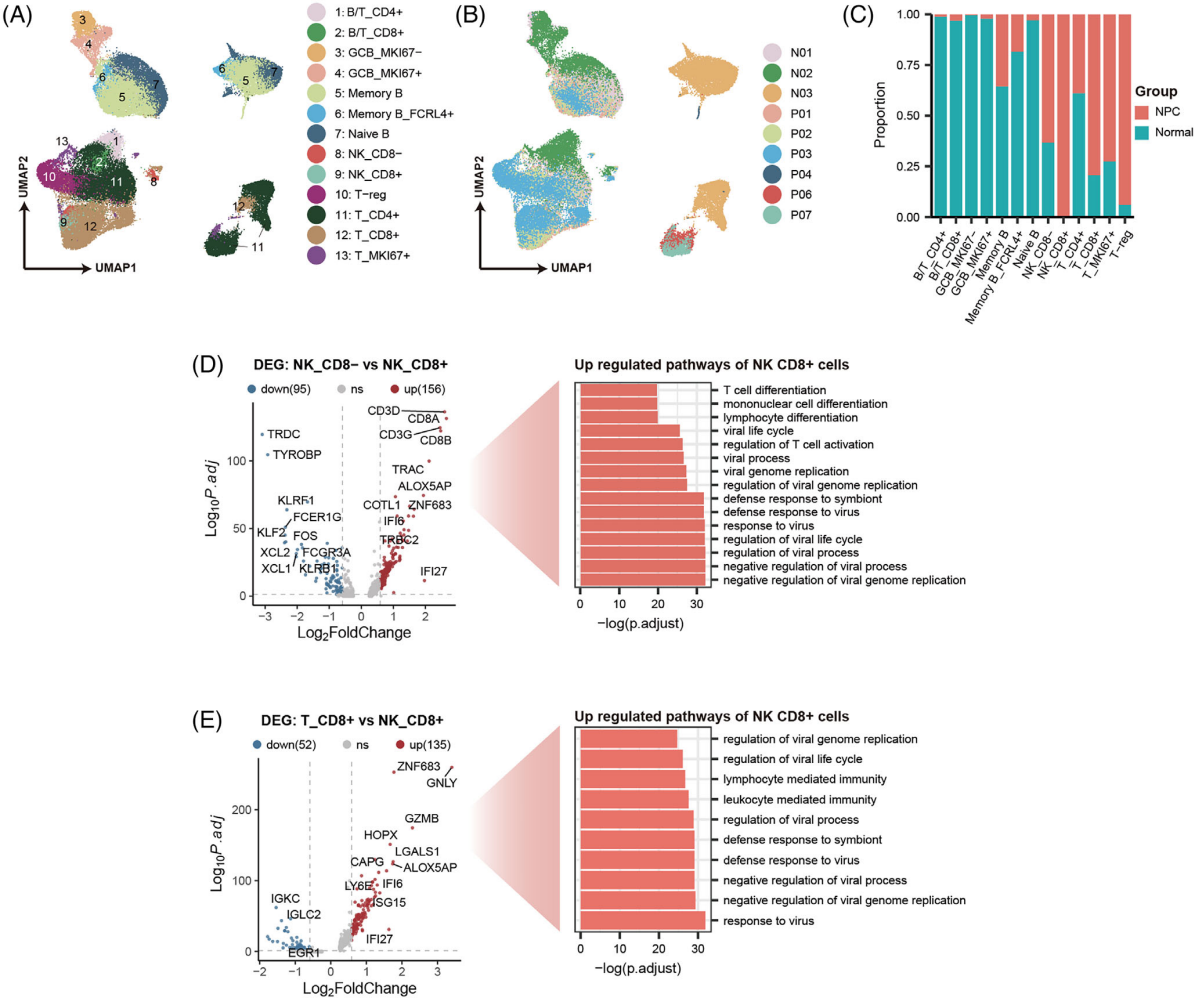
CD8+ NK cells enhanced antiviral functions. (a and b) UMAP plot of all lymphocytes coloured by annotated cell types and cell sample origin. (c) Stacked bar charts represent the proportion of each lymphocyte subcluster that is of tumour tissue origin and normal tissue origin (red: from nasopharyngeal carcinoma [NPC] tissues; cyan: from normal tissues). (d) Differentially expressed genes between CD8– NK cells and CD8+ NK cells, and their Gene Ontology (GO) enrichment analysis results. (e) Differentially expressed genes between CD8+ T cells and CD8+ NK cells, and their GO enrichment analysis results.

Using both scRNA and snRNA data, we annotated all lymphocytes into 13 subclusters based on differential expression of marker genes (Figures 5A,B and S4C). While most subclusters of lymphocytes have been reported in previous NPC scRNA studies,^5^, ^6^, ^7^, ^8^ we identified two new cell types: B/T cells and CD8+ NK cells. B/T cells express both B‐ and T‐cell markers (*MS4A1*, *CD79A*, *CD3D*, *CD4*, *CD8A*) and are primarily found in normal tissue samples (Figure 5c). Given the stringent quality control measures we employed during data preprocessing, as well as the reasonable feature and count number observed in B/T cells (Figure S4a–c), we ruled out the possibility that these cells were doublets. CD20+ T cells have been reported in several studies^17^, ^18^, ^19^ and are associated with chronic inflammation, but their relationship with nasopharyngeal or NPC tissues remains unclear.

CD8+ NK cells are a distinct cell population that we identified in the NPC TME, as they were not found to in normal nasopharyngeal tissues (Figure 5C). Although CD8+ NK cells have not been previously reported in other NPC studies, they have been observed in other diseases associated with HIV and EBV infections.^20^, ^21^, ^22^ DEG analysis and the DEG enrichment analysis (Figure 5D) of CD8+ NK cells and CD8– NK cells revealed that CD8+ NK cells significantly upregulated the genes related to antiviral pathways. Similar results were obtained when we included CD8+ T cells in the analysis (Figure 5E), indicating that CD8+ NK cells have a notably enhanced antiviral capacity compared to typical NK cells and CD8+ T cells.

## 3 DISCUSSION

NPC has a high rate of metastasis and recurrence, which is one of the main causes of poor prognosis. Immune escape of in situ tumour cells may lead to distant metastasis and recurrence of NPC. Although existing studies of NPC have begun to use single‐cell sequencing technology to study the heterogeneity of the TME and have relatively completely deciphered the cellular composition of the NPC TME, we have found that the application of scRNA‐seq technology in NPC tissue significantly underestimates the proportion of epithelial cells/tumour cells, and in many samples, it does not capture significant clusters of epithelial/tumour cells, which hinders the heterogeneity study of malignant cells in NPC, including immune escape features. There are clear differences between the scRNA and snRNA techniques in displaying the NPC TME. scRNA is more effective in capturing immune cells in the NPC TME, while snRNA is more effective in capturing epithelial/malignant cells. In this study, we used multiple single‐cell methods, including scRNA‐seq, snRNA‐seq and IMC, to effectively capture the epithelial and tumour cells in the NPC TME, filling the gap in the study of malignant cell heterogeneity in previous research.

Based on our analysis of multiomics single‐cell data, we identified three immune escape patterns of malignant cells in NPC.

First, some malignant cell clusters downregulated MHC molecule expression. In the immune response to tumours, MHC molecules are crucial for identifying and presenting antigens, guiding the immune system to attack foreign substances or abnormal cells. Dysfunctional MHC molecules are considered one of the important mechanisms of tumour immune escape. We found that there is heterogeneity in the expression of MHC molecules among tumour cell clusters, with a significant decrease in expression in some malignant cell clusters, which may allow them to initially escape immunity. Malignant cells with low MHC expression had significantly higher cell proliferation capacity than those with high MHC expression.

Second, we identified a fibroblast‐like malignant cell. This cell cluster was found in all snRNA‐seq samples and was characterised by the expression of the fibroblast marker COL1A1, and chromosomal copy number abnormalities, which is one of the malignant cell characteristics. This is a type of malignant cell that distinguishes itself from non‐malignant CAF cells. By analysing markers related to the EMT process, we found that the expression of epithelial polarity molecules in fibroblast‐like malignant cells was significantly reduced compared to other malignant cell clusters. We speculate that the EMT process is a potential pathway for this differentiation from normal malignant cells to fibroblast‐like malignant cells. In addition, compared to epithelial‐like tumour cells, we found that the MHC molecule expression in fibroblast‐like malignant cells was further reduced, and the expression of oncogenic Igs was upregulated, both of which have been reported in previous studies to be associated with increased immune escape ability. Previous studies have shown that CSCs possess the characteristics that include Ig expression,^16^ EMT features^23^ and immune evasion^24^. Considering fibroblast‐like malignant cells also have these features, we speculate that they are potential tumour stem cells, but further wet experiments are needed to confirm this.

Third, the tumour nest utilises hyperplastic epithelial cells to physically reduce immune infiltration, indirectly enhancing the immune escape ability of tumour cells in the tumour nest. IMC imaging showed that there are non‐malignant hyperplastic epithelial cells in the NPC TME, and immune cells can hardly infiltrate the cell layer composed of these cells. Furthermore, we observed that hyperplastic epithelial cells showed increased proliferation only when they were adjacent to non‐tumour areas, which was indicated by the expression of the proliferation‐associated marker Ki67+. This means that the hyperplastic areas cannot maintain the boundary with the tumour nest and can only proliferate towards non‐tumour areas, making itself a pioneer and accomplice in the expansion of the tumour nest. The tumour nest can invade the non‐immune infiltrated hyperplastic areas.

In addition, we discovered a new subtype of immune cells: CD8+ NK cells, which were not previously identified in single‐cell studies of NPC. CD8+ NK cells are rarely found in other cancers and are not found in non‐malignant nasopharyngeal microenvironments. Enrichment analysis showed that compared to CD8– NK cells and CD8+ T cells, CD8+ NK cells significantly increased antiviral function, demonstrating their special role in the TME of NPC.

There are still limitations in this study. First, there is a lack of wet‐laboratory validation. The complex TME of NPC, including EBV infection and immune infiltration, shapes the heterogeneous malignant cell characteristics. This presents a significant challenge for wet‐laboratory validation through in vitro and animal experiments; hence, we did not conduct wet‐laboratory validation. However, further experimental validation of the immune escape characteristics of malignant cells in NPC and CD8+ NK cells is important and worth further research. Second, there is still a lack of research on the correlation between EBV and immune escape characteristics of malignant cells, which is as a result of the low count of EBV virus detected in our sequencing data (Figure S5). Enhancing the detection sensitivity of target viral transcripts during library preparation is necessary for future single‐cell studies related to NPC or other viral‐associated research.

In summary, our study has provided new insights into the heterogeneity of immune escape in situ malignant cells in NPC. Our findings may help develop new therapies targeting immune escape mechanisms and ultimately improve clinical outcomes for patients with NPC.

## 4 METHODS

### 4.1 Patients and samples

In this study, seven patients with primary NPC and two patients with chronic nasopharyngitis were enrolled (Table S1). All tissue samples were collected by clinical biopsy and assessed by at least two experienced pathologists. This study was approved by the Ethics Committee of Peking University Shenzhen Hospital and Shenzhen People's Hospital. Written informed consent was obtained from all patients.

### 4.2 Single‐cell suspension preparation

On ice, use phosphate‐buffered saline (1× PBS, Ca^2+^ and Mg^2+^ free) to clean the blood and mucus on the fresh tissue samples after the biopsy. Cut the tissue samples into pieces and add 1 mL digestive enzyme mixture containing 1000 μL Hank's balanced salt solution (HBSS) buffer + 1 μL DNase I (2000 U/mL) + 20 μL collagenase type IV (10%) + 5 μL hyaluronidase (HAase) (10%) + 10 μL dispase (5%) + 1 μL CaZnMg buffer. Use water bath heating to dissociate the tissue at 37°C for 40 min. Shake the centrifuge tube every 3 min. Add 3 mL 1× HBSS to each sample, filter the solution with 70 μm cell strainer, centrifuge the filtered solution horizontally at 300 *g* for 5 min at 4°C and discard the supernatant. Add 1 mL precooled 1× PBS buffer to centrifugal sedimentation, then add 3 mL 1× red blood cell lysis buffer (RCLB) with sufficient mixing and let stand at room temperature for 2 min. Resuspend the cells with 5 mL 1× PBS buffer and centrifuge horizontally at 300 *g* for 5 min at 4°C, repeat twice to remove the cell debris. Add 2 mL precooled 1× PBS buffer and fully mix, then use a 40 μm cell strainer to filter the suspension, wash with 3 mL 1× PBS buffer, centrifuge the filtered solution horizontally at 300 *g* for 5 min at 4°C and discard the supernatant. The cells were stained with trypan blue and the cell concentration and viability were counted using the microscope counting chamber (hemocytometer). Use 1× PBS buffer to dilute the solution to the target concentration of 1000−2000 cells/μL.

### 4.3 Single‐nucleus suspension preparation

On ice, use 1× PBS (Ca^2+^ and Mg^2+^ free) to clean the blood and mucus on the fresh tissue samples after the biopsy and dry the tissue sample by sterile blotting paper. Then, cut the tissue samples into pieces, put the tissue into cryovials and quick‐freeze cryovials in liquid nitrogen for 5 min. Store the cryovials in the liquid nitrogen for long‐term storage. Transfer the tissue from cryovials to Dounce tissue grinder. Thaw the tissue at room temperature, then aspirate 500 μL lysis buffer (0.25 M sucrose, 5 mM CaCl_2_, 3 mM MgCl_2_, 10 mM Tris–HCl pH 8.0, 1 mM Dithiothreitol, 0.1 mM ethylenediaminetetraacetic acid (EDTA), 1× protease inhibitor, 1 U/μL RiboLock RNase inhibitor) to the tissue sample tube and submerge the tissue. First, use the smaller pestle press for 15−20 times vertically. Then, use tighter pestle press for 5−10 times vertically until the tissue is homogenised. Add 700 μL nucleus wash buffer (0.04% (bovine serum albumin [BSA], 0.2 U/μL RiboLock RNase inhibitor, 500 mM mannitol, 0.1 mM Phenylmethylsulfonyl fluoride (PMSF) protease inhibitor, dissolved in 1× PBS), inverting the tube and shake five times for blending. Use a 70 μm cell strainer to filter the homogenate. Add 1 mL 50% iodixanol solution to the filtrate, pipetting up and down gently until fully mixed. Preparation of gradient solutions: add 1 mL 33% iodixanol solution to the bottom of the 15 mL centrifuge tube. Then, add 2 mL 30% iodixanol solution along with the wall of the centrifuge tube. Slowly add 2 mL of the nucleus contained Iodixanol solution along with the wall of the centrifuge tube to the liquid surface of 30% iodixanol solution. Centrifuge at 10 000 *g* for 20 min at 4°C. Transfer 1 mL nucleus layer to a new 15 centrifuge tube. Take 2 mL nucleus wash buffer and invert the tube and shake 10 times for blending. Centrifuge at 500 *g* for 8 min at 4°C. Discard the supernatant. Repeat this step twice. Use a 40 μm cell strainer to filter the nucleus suspension. Centrifuge at 500 *g* for 5 min at 4°C. Discard the supernatant and then add another 100 μL nucleus wash buffer to resuspend the nucleus. Stain cells with trypan blue and count the cell concentration and viability using the microscope counting chamber (hemocytometer). The target concentration is 700−1200 cells/μL, use 1× PBS buffer to dilute the solution if the nucleus concentration is too high.

### 4.4 Library construction and sequencing

Single‐cell suspension or single‐nucleus suspension was used for library construction, and 8000−12 000 cells or nuclei were loaded per sample. Following the manufacturer's instructions, the libraries were constructed by using Chromium Single Cell 3′ Reagent Kits v3 (10× Genomics). The cDNA libraries were sequenced on the Illumina 10× Genomics Chromium platform (Illumina Novaseq 6000).

### 4.5 scRNA‐seq and snRNA‐seq data quality control

FASTQ files were processed by CellRanger (version 5.0.1), and the reference for CellRanger processing contains the human genome (GRCh38) and EBV genome (NC_007605.1). For scRNA‐seq data, intronic reads were excluded; for snRNA‐seq data, intronic reads were included. Seurat R package (version 4.1.1) was used to convert the raw gene expression matrix into Seurat object. Doublets were inferred by the R package ‘scDblFinder’ (version 1.11.3). Cells with <251 expressed genes, <501 total counts, >20% mitochondrial counts or <.8 log10GenesPerUMI were considered as low‐quality cells and were removed from downstream analysis.

### 4.6 CNV inferring

CNV inferring was processed by using the R package ‘inferCNV’ (https://github.com/broadinstitute/inferCNV). We performed CNV inferring for cell types containing malignant cells, such as fibroblasts and epithelial cells. For the scRNA data, we used target type cells from N01 and N02 sample sources as reference cells. For the snRNA data, we used the target type cells from the N03 sample source as the reference cells. Considering tumour heterogeneity, CNV inferring was performed separately in each sample dataset. The cutoff parameter was set to 0.1, as it works well for 10× Genomics data. Other parameters used in this analysis follow the default setting.

### 4.7 Cell signature scoring

The different signatures of cells were evaluated and scored using the Seurat function ‘AddModuleScore’. The genes included in each feature set can be found in Table S2. EMT score based on E.EMT score and M.EMT, as shown in Figure S3E.

### 4.8 Gene set enrichment analysis

We used the R package ‘clusterProfiler’ for gene set enrichment analysis (GSEA). Genes that had the absolute value of log2 fold‐change (FC) of the average expression between the two groups over 0.5 (abs[avg_log2FC] > 0.5) were defined as DEGs for GSEA. Other parameters followed the default settings.

### 4.9 Transcriptomics data‐based cell–cell interaction inferring

In this project, the ‘CellChat’ R package (version 1.1.3) was used for revealing the potential interaction and communication between different cell types. Human databases, including Secreted Signalling subset, ECM‐Receptor subset and Cell–Cell Contact subset, were chosen as the ligand–receptor interaction databases.

### 4.10 IMC antibody staining and acquisition

The antibodies conjugated with metals were diluted in 0.5% BSA and stored at 4°C for staining. Slide‐tissue sections were baked at 65°C for 1 h. Tissue sections were deparaffinised with two washes of 100% fresh‐xylene and then rehydrated with successive washes with ethanol 100% (2×), 95% (2×), 85% (1×), 70% (1×) and distilled water. The sections were then immersed in antigen retrieval buffer (Tri‐EDTA buffer, pH 9.0; Abcam, Cambridge, UK), incubated at 96°C for 30 min, and cooled down to room temperature at room temperature. Slides were washed with distilled water (1×) and Dulbecco's PBS (DPBS, 1×) for 10 min each and then washed with distilled water for 8 min on an orbital shaker. Next, the tissue was blocked with a blocking buffer for 30 min at 25°C −30°C (Superblock Blocking Buffer in PBS; Thermo Fisher Scientific, Waltham, MA, USA) in a humid chamber. After blocking, the antibody mix was then applied and incubated overnight at 4°C in a humid chamber. After overnight incubation, the slides were washed on an orbital shaker for 5 min in wash buffer. Thereafter, tissue sections were successively washed with 0.2% Triton X‐100 (2×) and DPBS (2×) on an orbital shaker. Subsequently, slides were incubated with intercalator‐Ir diluted 1:400 in DPBS for 30 min at room temperature. After incubation, the sections were washed with distilled water for 5 min. Finally, the slides were dried under airflow for 5 min and stored at 4°C until ablation.

The Hyperion mass cytometry system (Fluidigm Corporation, South San Francisco, CA, USA) was autotuned using a three‐element tuning slide according to the tuning protocol provided by the manufacturer. Then, ROIs were selected based on consecutive tissue sections stained with HE. ROIs of 1000 × 1000 μm were ablated and acquired at 200 Hz for approximately 2 h. Data were exported as MCD files and visualised using the Fluidigm MCD™ viewer. To better separate the antibody signal and noise, each marker was visually inspected, and a minimum signal threshold of one or two dual counts was set in the Fluidigm MCD™ viewer.

### 4.11 IMC data analysis

Data from Hyperion mass cytometry system were converted to TIFF format using the Fluidigm MCD™ Viewer for data visualisation. The preprocessing of IMC images for individual cell segment followed the protocol of ‘ImcSegmentationPipeline’ (https://github.com/BodenmillerGroup/ImcSegmentationPipeline). We processed image segmentation and cell measurement with CellProfiler (version 4.2.1) and Ilastik (version 1.3.3) software tools. The downstream analysis of preprocessed IMC images, including but not limited to: object construction, dimension reduction and visualisation, cell clustering and phenotyping, and spatial analysis, was based on the ‘imcRtools’ R packages.^25^

## CONFLICT OF INTEREST STATEMENT

The authors declare they have no conflicts of interest.

## Supporting information

Supporting Information

## Data Availability

The raw sequence data reported in this paper have been deposited in the Genome Sequence Archive in National Genomics Data Center, China National Center for Bioinformation/Beijing Institute of Genomics, Chinese Academy of Sciences (GSA‐Human: HRA003340) that are publicly accessible at https://ngdc.cncb.ac.cn/gsa‐human. The R codes can be found in https://github.com/Linxiaobuskiy/NPC.
