# Supplementary material for: Single‐cell analysis reveals the multiple patterns of immune escape in the nasopharyngeal carcinoma microenvironment
Source: Clin Transl Med. 2023 Jun 22;13(6):e1315. doi: 10.1002/ctm2.1315 (PMC10288070; doi:10.1002/ctm2.1315)
Supplement: Supplementary file 1 — Supporting Information [file CTM2-13-e1315-s001.docx]

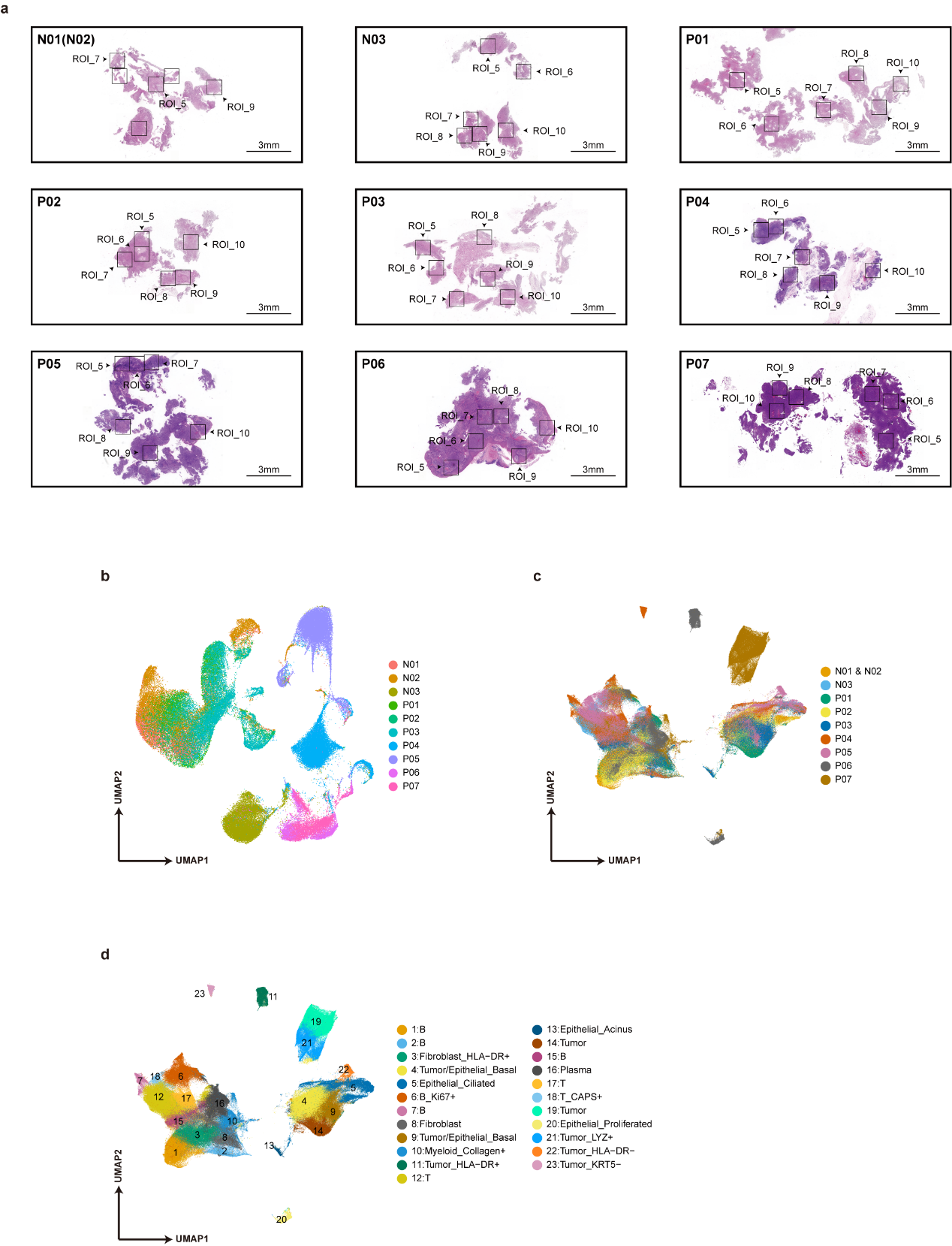


**Supplementary Fig. 1: The ROIs on paraffin sections used for IMC and the multiomics Atlas colored by different groups.**

**a** The ROIs on paraffin sections used for IMC, labelling on HE stained serial paraffin sections.

**b** UMAP plot of all single cells obtained in scRNA/snRNA-seq coloured by samples.

**c** UMAP plot of all single cells obtained in IMC images coloured by samples.

**d** UMAP plot of all single cells obtained in IMC images coloured by cell types.


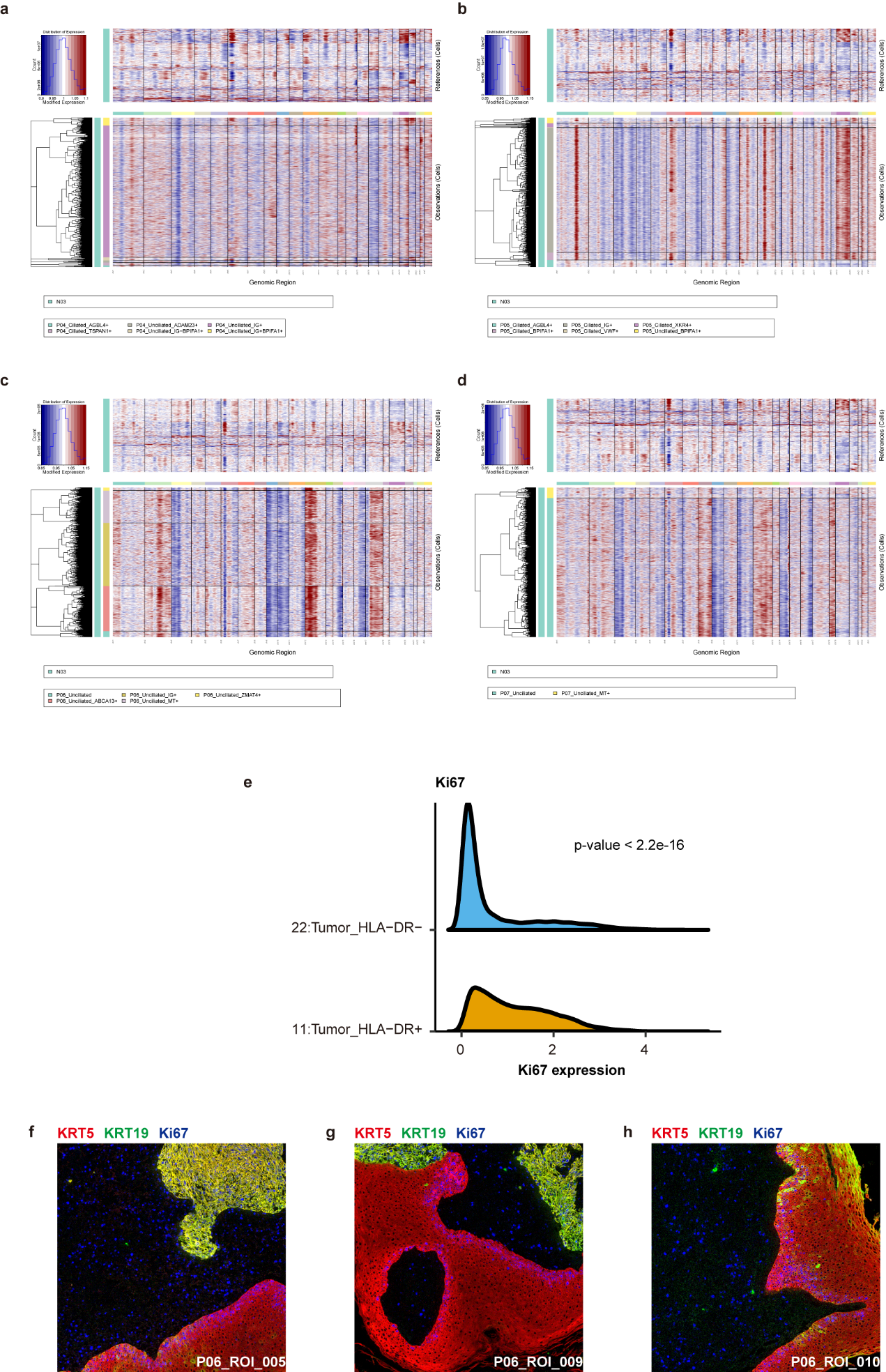


**Supplementary Fig. 2: Heterogeneity of tumour cells and epithelial cells in the genome and proteome.**

**a-d** The CNV inferring results of epithelial cells in P04-P07 samples.

**e** The RidgePlot of Ki67 expression in HLA-DR- tumour cells and HLA-DR+ tumour cells.

**f-h** The heterogeneity between tumour cells and hyperplasia cells. IMC images numbered P06_ROI_005, P06_ROI_009 and P06_ROI_010. Pseudostaining for KRT5 (red), KRT19 (green), and Ki67 (blue) in cells. Each image size is 1 mm square.


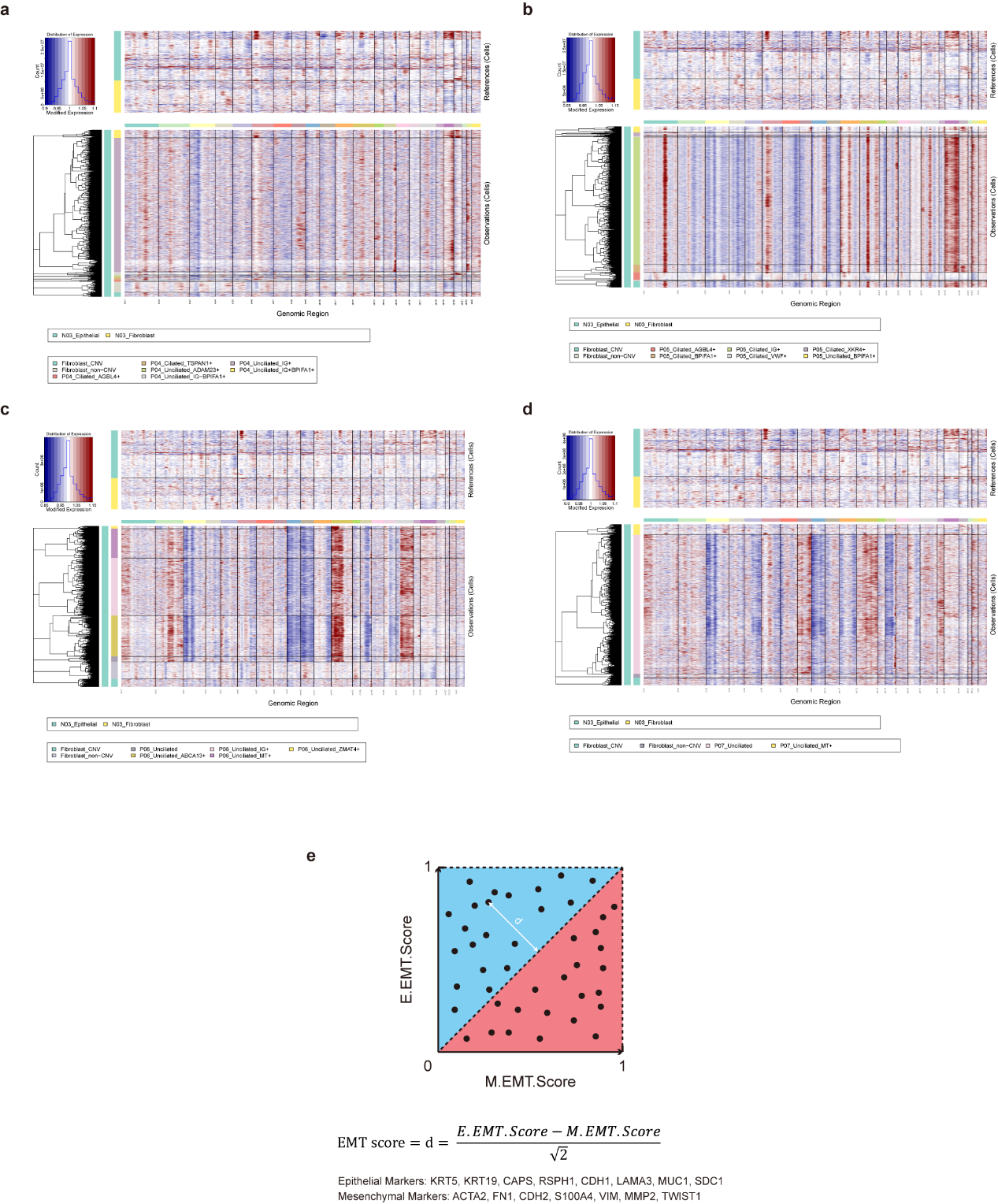


**Supplementary Fig. 3: The CNV inferring results of fibroblast-like malignant cells in P05-P07 samples.**

**a-d** The CNV inferring results of fibroblast-like malignant cells in P04-P07 samples individually.

**e** Schematic representation of the EMT scoring principle. Areas representing epithelial polarisation are coloured blue and areas of mesenchymal polarisation are coloured red.


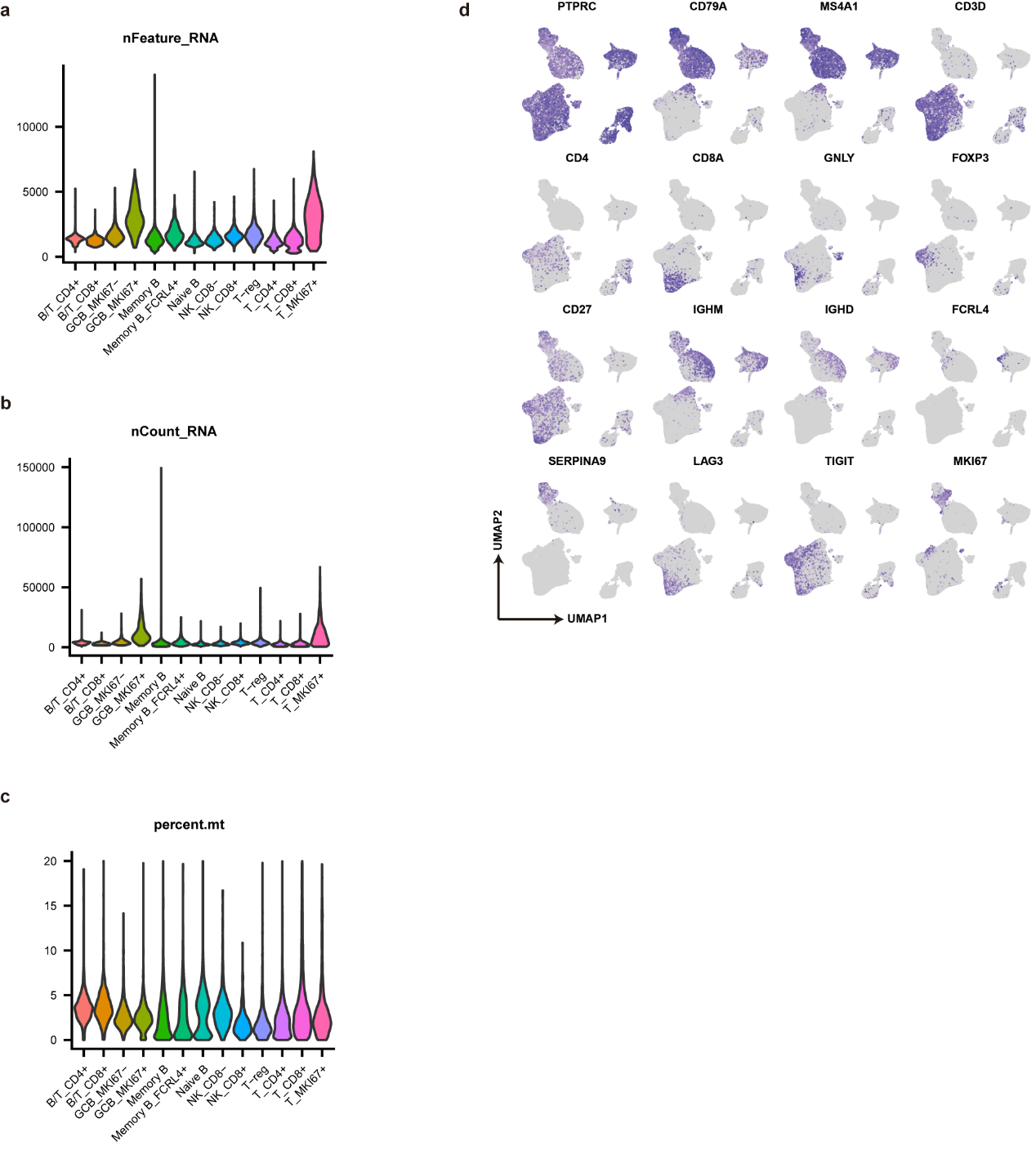


**Supplementary Fig. 4: The characteristics of lymphocytes in the NPC TME.**

**a** The number of unique genes detected in lymphocytes, grouped by cell clusters and visualized by violin plots.

**b** The total number of molecules detected in lymphocytes, grouped by cell clusters and visualized by violin plots.

**c** The mitochondrial genome reads percentage of lymphocytes, grouped by cell clusters and visualized by violin plots.

**d** The feature plot of marker genes. The marker expression from low to high is represented by grey to blue.


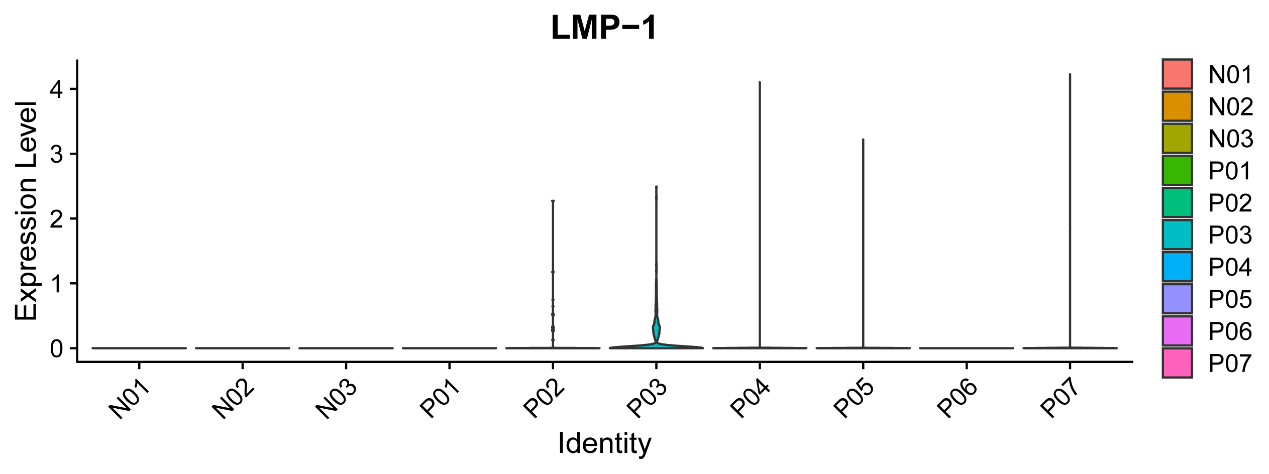


**Supplementary Fig. 5: The expression of LMP-1 in epithelial/tumour cells.**

The violin plot of the expression levels of the EBV virus gene LMP-1 in the epithelial/tumor cells across various samples.

**Supplementary Table. 1: Information of the patients enrolled in this project.**

| PatientID | SampleID | Gender | Age | Subtype | TNM | Sequencing method | Patient Origin |
| --- | --- | --- | --- | --- | --- | --- | --- |
| 1 | N01 | F | 50 | non-malignant |  | single-cell | Shenzhen |
| 1 | N02 | F | 50 | non-malignant |  | single-cell | Shenzhen |
| 2 | N03 | M | 36 | non-malignant |  | single-nucleus | Shenzhen |
| 3 | P01 | F | 48 | nonkeratinized/differentiated | T4aN2M0 | single-cell | Shenzhen |
| 4 | P02 | M | 34 | nonkeratinized/differentiated | T4N0M0 | single-cell | Shenzhen |
| 5 | P03 | F | 36 | nonkeratinized/differentiated | T4N2M0 | single-cell | Shenzhen |
| 6 | P04 | M | 37 | nonkeratinized/differentiated |  | single-nucleus | Shenzhen |
| 7 | P05 | M | 48 | nonkeratinized/undifferentiated | | single-nucleus | Shenzhen |
| 8 | P06 | M | 33 | nonkeratinized/undifferentiated | | single-nucleus | Shenzhen |
| 9 | P07 | M | 34 | nonkeratinized/differentiated |  | single-nucleus | Shenzhen |

**Supplementary Table. 2: Genes used for cell signatures scoring.**

| **Groups** | **Genes** |
| --- | --- |
| E.EMT | KRT5, KRT19, CAPS, RSPH1, CDH1, LAMA3, MUC1, SDC1 |
| M.EMT | ACTA2, FN1, CDH2, S100A4, VIM, MMP2, TWIST1 |
| Hypoxia | HIF1A, EPAS1, HIF3A, SLC2A1, PDK1, HK1, HK2, HK3, GAPDH, ALDOA, ENO1, PGK1, PFKM, PFKP, PFKL, LDHA |
| Mitochondrion | MT-ND1, MT-ND2, MT-CO1, MT-CO2, MT-ATP8, MT-ATP6, MT-CO3, MT-ND3, MT-ND4L, MT-ND4, MT-ND5, MT-ND6, MT-CYB |
| CSC | CD44, PLAUR, THY1, PROM1, ALCAM, EPCAM, ALDH2, NANOG, POU5F1, CD24, ITGB1, TGA6, ITGB3, CD70, CXCR4, LGR5, PROCR, BMI1, NOTCH1, SOX2, LINGO2, LETM1, MSI2, AFP, SALL4, CLEC12A, HAVCR2, IL2RA, DPP4, CD33, KIT, IL3RA, IL1RAP |
| MHC-I | HLA-A, HLA-B, HLA-C, HLA-E, HLA-F, HLA-G |
| MHC-II | HLA-DRB5, HLA-DQA2, HLA-DQB2, HLA-DOA, HLA-DOB, HLA-DMA, HLA-DMB |
| MHC | MHC-I genes and MHC-II genes |
| M1 | CD80, CD86, FCGR1A, FCGR2B, FCGR3B, TLR2, TLR4, TNF, IL1A, IL1B, IL6, IL12A, IL12B, IFNA1, IFNA2, IFNA4, IFNA5, IFNA6, IFNA7, IFNA8, IFNA10 IFNA13, IFNA14, IFNA16, IFNA17, IFNA21, IL1R1, CXCL9, CXCL10, CXCL11, CCL2, CCL3, CCL4, CCL5, CXCL8, CCR7); |
| M2 | MRC1, CD163, CD68, MSR1, SCARB1, SCARB2, CD36, CD14, FCER2, IL1RN, IL10, IL1R2, CCL17, CCL22, CCL24, CCL18, CCL16, CCR2, CXCR1, CXCR2, TGFB1, TGFB2, TGFB3, IL4 |
